# Validation of the Coffee Cuality™ Method for the Expert Assessment of Coffee Sensory Quality

**DOI:** 10.3390/foods15040678

**Published:** 2026-02-12

**Authors:** Jean-Xavier Guinard, Lik Xian Lim, Benjamin Elliott, Andrew Cotter

**Affiliations:** Department of Food Science and Technology, University of California, Davis, Davis, CA 95616, USA; lxlim@ucdavis.edu (L.X.L.); btelliott@ucdavis.edu (B.E.); arcotter@ucdavis.edu (A.C.)

**Keywords:** expert quality assessment, specialty coffee, sensory evaluation, sensometrics, roast level

## Abstract

The Coffee Cuality Method provides a comprehensive assessment of the sensory quality of coffee that includes an overall quality rating, just-about-right (JAR) scaling of select attributes, check-all-that-apply (CATA) selections from a list of sensory and holistic attributes and open comments. We validated the method with 56 expert coffee tasters by comparing Coffee Cuality with their customary method (i.e., SCA’s, Q-grading or company’s own) for the evaluation of the sensory quality of 12 specialty coffees and commercial blends brewed with their preferred method (cupping, drip, pour over or espresso). A subset of 18 experts then participated in focus groups on the method. Quality mapping (principal component and cluster analyses of the quality ratings) showed consistency among the experts’ overall quality ratings regardless of brewing method, with the dark roasts rated systematically lower than the light- and medium-roasted coffees. Penalty analysis relating JAR ratings to quality scores showed that too dark of a roast or a beverage color and too low of an acidity had the largest (negative) impact on quality. The map of sensory and holistic attributes derived from CATA selections by correspondence analysis, and the word clouds of those selections showed which attributes drove quality ratings, positively or negatively. Focus groups with a subset of the experts suggested improvements to the evaluation protocol and scorecard. By deconstructing, documenting and justifying coffee quality ratings, Coffee Cuality offers a valuable alternative to conventional evaluation protocols.

## 1. Introduction

Even though the so-called central dogma in sensory evaluation [1] of the past century called for practitioners to stay away from quality judgments as they were too subjective and preference tainted, there is no question that the evaluation of sensory quality is needed and that it is best to turn to experts (i.e., individuals with experience and knowledge of possible defects, trueness to type or standards of (sensory) identity and desirable sensory features for the product category) to evaluate it. So rather than dismissing sensory quality ratings by experts as we were taught, we choose to focus on how current practices and tools could be improved.

**Figure 1:**
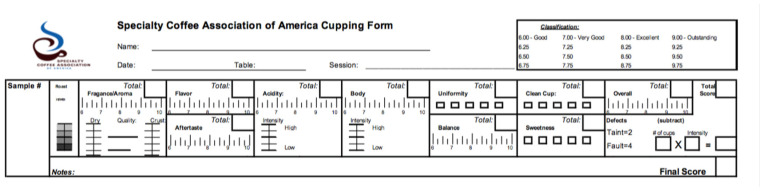
The Specialty Coffee Association of America (now SCA) cupping form with its various components and final score out of 100 points.

Since 2003 for Arabica Coffee and 2010 for Robusta Coffee, the sensory quality of coffee, particularly that of specialty coffee, has long been evaluated through a cupping protocol and a 100-point scoring scheme, or Q-grading, that combines ratings of various aspects of the coffee on different scales including fragrance/aroma, flavor, aftertaste, acidity, body, balance, uniformity, sweetness, clean cup, overall, and the total score as shown on the original Specialty Coffee Association of America (SCAA) coffee cupping form in Figure 1 [2,3]. Such quality ratings have been the standard for the valuation and trading of green coffee and the judging of coffees at competitions [4,5,6,7]. A coffee scoring above 80 is considered a specialty coffee, thus bringing it higher value in the trade [8]. And the SCA/Q-grading protocols have sometimes been used in research on the drivers of coffee quality as a substitute for, or in combination with sensory evaluation and/or consumer testing methods [9,10,11].

The limitations of such a scoring system have long been recognized, however, chief among them the mixing of very different constructs such as intensity, quality, and presence/absence of specific attributes and defects; their subjectivity; and the use of odd scales to rate them (Figure 1) [12]. Practically, we observed that so-called Q graders (those who are trained to rate the quality of coffee with this system) usually come up with an overall score and then fill in the various categories on the scoring sheet to add up to that score. Regardless, this traditional approach provides limited justification for the score received by the coffee. Was a coffee rated high because it had the perfect acidity (and what would that be)? Did a defect penalize the coffee, and by how much? Was the aroma noteworthy, or was it the taste of the coffee that weighed heavily into its score? And most importantly, how could coffee growers and roasters adjust the sensory profile of their coffee to improve its sensory quality based on the score it received?

We would be remiss not to mention the alternative of replacing the sensory coffee quality assessment methods altogether with objective, instrument-based approaches [13]. Indeed, the coffee analytical chemistry literature is replete with examples of successful identification and measurement of markers of coffee sensory quality [14,15,16,17,18,19]. And artificial intelligence could be the tool that affords us the ability to integrate such multidimensional and complex data into a measure of quality. But for now, a sensory evaluation approach is warranted.

In 2020, we introduced and trademarked the Coffee Cuality^TM^ Method for the assessment of the sensory quality of coffee by experts [20,21,22,23]. The aim at the time was to improve on the methodology used by coffee grading organizations by adapting modern sensory evaluation and consumer testing methods (i.e., just-about-right scaling and check-all-that-apply) [24,25,26,27] and sensometrics tools (i.e., factor analysis, classification and regression methods) to coffee quality assessment. Could we create a scorecard that would deconstruct, document and justify the quality rating that a coffee would receive from experts? For that purpose, we extended the approach we use in consumer testing to explain the hedonic rating that a consumer gives to a product on the nine-point hedonic scale. By adding just-about-right (JAR) scaling of key sensory attributes for the product, by having the consumer use a check-all-that-apply (CATA) task to describe the sensory and holistic attributes of the product, and finally by commenting on what specifically the consumer liked and then disliked about the product, we could then use a battery of statistical tools to uncover the positive and negative drivers of liking for that product and consumer, that is, deconstruct, document and justify that hedonic rating. We extended that methodological approach to quality ratings by experts, and that combination of quality ratings on a 100-point scale, JAR ratings, CATA selections and open comments became Coffee Cuality. Just as they allowed us to understand and justify consumer liking, those JAR ratings, CATA selections and open comments, analyzed with the proper multivariate statistical tools, are now used to document and justify what experts view as quality, as expressed in their quality ratings of a set of coffees. Other protocols that include some of the same descriptive (CATA) or affective (JAR) elements of Coffee Cuality have since been developed and are in use today. They are the SCA’s Coffee Value Assessment (CVA) protocol [28] and The Espresso Protocol (TEP) [29].

The objective of this research was to validate the Coffee Cuality Method by having coffee experts rate a set of 12 coffees with their customary method (SCA’s, Q grading, or company’s own) and then with the Coffee Cuality Method. We then compared the ratings and the outcomes of the two methods, Our main hypothesis was that the two methods would produce the same quality ratings and discriminate equally among the coffees. Secondary hypotheses were that the Coffee Cuality Method would then provide a full documentation and justification of the quality ratings generated by the experts and that it would validate their expertise by demonstrating their ability to discriminate among the coffees and their alignment with other experts in rating quality.

## 2. Materials and Methods

### 2.1. The Coffee Cuality Method

**Figure 2:**
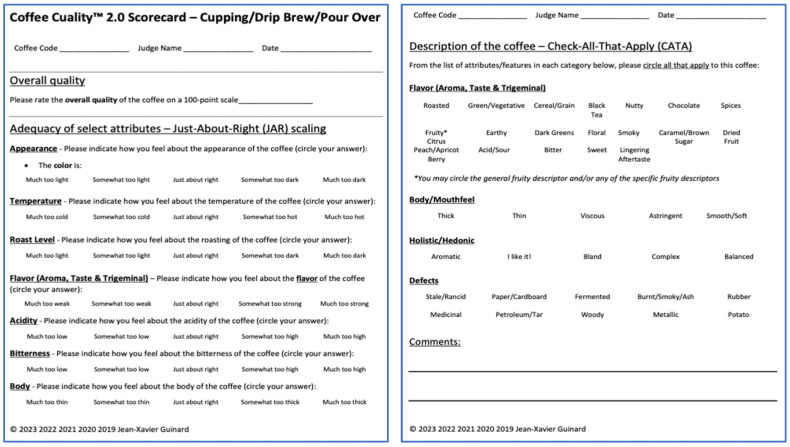
Coffee Cuality™ 2.0 scorecard for cupping, drip or pour over coffee.

Coffee Cuality consists of an overall quality rating on a 100-point scale and a battery of other measures meant to deconstruct and explain that overall quality rating. First, the adequacy of key sensory attributes for the type of coffee being scored is rated on just-about-right (JAR) 5-point scales that typically go from “much too low” to “much too high”, with the “just-about-right” category as the midpoint. We note here that this is different from rating the intensity of those attributes. Indeed, those indicate whether, from an expert’s perspective on sensory quality, those attributes are present at the right level or not. Second, a check-all-that-apply (CATA) list of sensory and holistic attributes is used by the expert to “describe” the sensory properties of the coffee, by checking those attributes that apply to the coffee. Because many attributes make up the sensory profile of coffee, we elected to move away from the generally accepted best practice of working with a list of 15 to 20 attributes and instead to break those down into various categories—of flavor (aroma, taste and chemesthesis), body/mouthfeel, hedonic/holistic and defects, with 5 to 21 attributes in each category. And finally, an open comments section invites the experts to comment on what made the coffee a high- or a low-quality one. The Coffee Cuality^TM^ 2.0 scorecard for cupping/drip brew/pour over is shown in Figure 2. And those for espresso and cold brew are shown in Supplementary Figures S1 and S2.

When used with multiple experts, Coffee Cuality then applies a suite of statistical tools to analyze the overall quality score and then justify it. First, mean quality ratings are computed and compared with a multiple mean comparison test such as Fisher’s least significant difference (LSD). Then, quality mapping and quality clustering analyses [30] are run to determine how each expert viewed sensory quality across the coffees (with each expert’s quality vector representing his/her main quality direction across the coffees) and to examine their degree of alignment on the concept of quality. Those analyses extend the concept of preference mapping and clustering for consumer hedonic ratings to quality ratings by experts [30]. The quality mapping analysis is a principal component analysis of the matrix of quality ratings by the experts across the coffees, and the quality clustering analysis is a cluster analysis of the same (but flipped) matrix. Distributions of JAR ratings for the coffees are displayed on bar graphs for each JAR attribute, and those distributions are compared using Stuart–Maxwell frequency and McNemar tests for comparison purposes, but more importantly, a penalty analysis is performed to show how a JAR attribute being judged too low or too high penalizes the overall quality score. CATA selections are analyzed by correspondence analysis to produce a biplot with both coffees and attributes that is essentially a sensory (and holistic) map of the coffees. A penalty/lift analysis is then performed on those CATA selections to determine positive and negative drivers of quality. Finally, word cloud analyses are performed on the CATA selections on one hand and on the open comments for each coffee on the other.

### 2.2. Validation of the Coffee Cuality Method

To validate the Coffee Cuality Method, we had 56 coffee experts evaluate 12 specialty or commercial coffees using their habitual quality evaluation method (i.e., SCA’s, Q-grading, or company’s own) first and then with the Coffee Cuality Method.

#### 2.2.1. Coffees

To cover a wide range of sensory profiles and quality, the experimental design for this research included 12 specialty or commercial coffees from different origins and with different degrees of roasting and a practice coffee. A description of the coffees can be found in Table 1. To preserve their freshness, an important driver of quality [31], the 13 coffees were shipped overnight to the experts as whole beans in airtight Ziploc bags (S. C. Johnson & Son, Inc., Racine, WI, USA) coded with 3-digit codes.

#### 2.2.2. Experts

Over seventy coffee experts from the specialty coffee industry were invited to participate in this study. Fifty-six (56) completed it. They were deemed “experts” because they were certified Q-graders or because the sensory evaluation of coffee was an integral part of their job description. They were asked to complete their evaluations of the coffee within one week of their receipt.

This study was approved by the Institutional Review Board of the University of California, Davis (protocol number 1082569-2).

#### 2.2.3. Quality Evaluation Protocol

Experts were instructed to brew the coffees using their preferred grind size and brewing method (i.e., cupping, drip or espresso) and then to evaluate them in the (randomized) order indicated in their ballot, first with their usual quality assessment method, and then with Coffee Cuality, starting with a quality rating on a 100-point scale for both methods (Figure 2). For their coffee preparation, most of the experts chose to follow the SCA’s brewing protocol for cupping [28]. Experts were instructed to scan and email their completed ballots to the experimenters.

#### 2.2.4. Focus Groups

Eighteen of the experts who rated the coffees also participated in three (3) focus groups of 5 to 8 participants each, in which they were asked about their experience with the Coffee Cuality method and for suggestions on how to improve the testing protocol and scorecard and were then informed about the goals and purposes of the new method.

## 3. Results

### 3.1. Coffee Cuality vs. Conventional Method

The two methods—conventional or preferred and Coffee Cuality—produced the same overall quality ranking of the coffees and similar ratings, as shown in Table 2. But the Coffee Cuality ratings were more widespread, from 59.04 to 82.71 (compared with 66.30–79.60 with the conventional method) and yielded more significant differences among the coffees.

**Figure 3:**
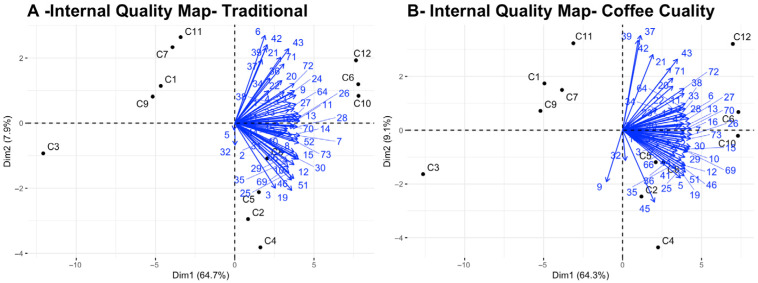
Internal quality maps derived from the experts’ traditional or habitual method of quality assessment (**A**) or Coffee Cuality (**B**), showing both the 12 coffees and the 56 experts’ quality vectors.

**Figure 4:**
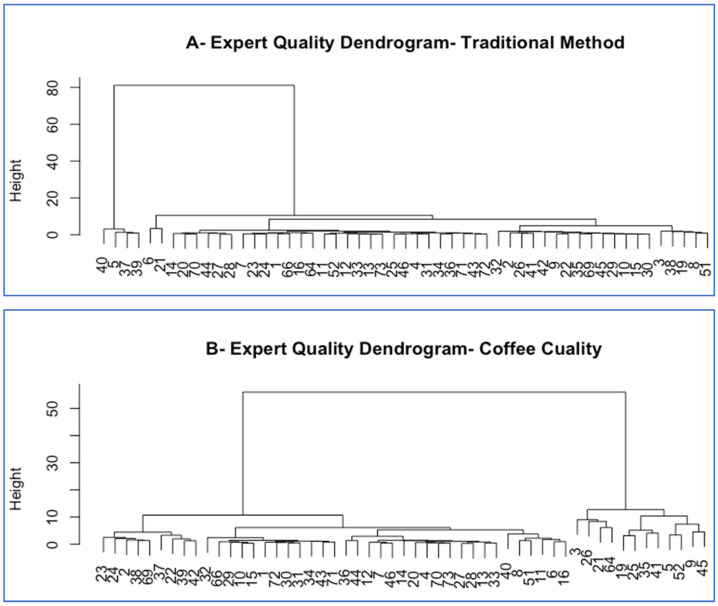
Expert quality dendrograms derived by cluster analysis from the matrices of expert quality ratings across coffees for the traditional or habitual method (**A**) and Coffee Cuality (**B**) (n = 56 experts, Numbers at the bottom of the dendrograms refer to the experts).

The quality maps generated by the traditional or preferred method and Coffee Cuality are shown in Figure 3A,B. Quality mapping is an extension of preference mapping whereby a matrix of quality ratings across products is analyzed by PCA [22], so that experts’ quality vectors are displayed along with the coffees in a PCA biplot. The quality maps for both methods are very similar again, in terms both of the direction of the quality vectors of the experts and of which coffees were judged to be of high quality (C8, C10, and C12) and which were rated lower (C1, C3, C7, C9 and C11). The agreement among the experts was slightly better with the experts’ conventional or preferred method than with Coffee Cuality, as shown by the slightly greater spread of angles in the quality maps/biplots and the smaller cluster of “different” experts (only 4 for the traditional or preferred method versus 12 for Coffee Cuality) based on hierarchical agglomerative clustering of the “Euclidean” distance measurements of the quality scores, using the “Ward” algorithm (Figure 4A,B).

**Figure 5:**
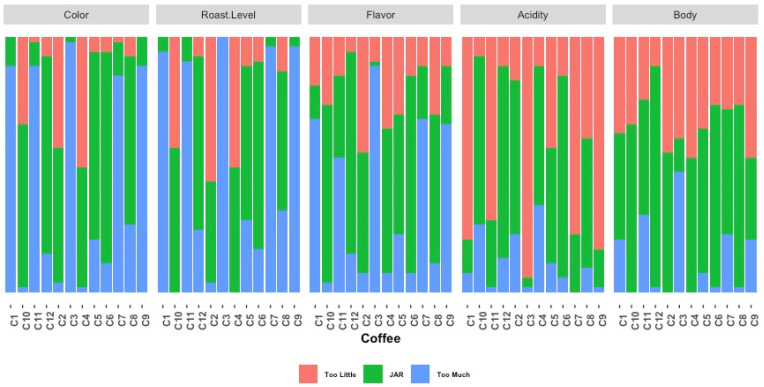
Just-about-right (JAR) rating distributions for color, roast level, flavor, acidity and body across the 12 coffees from the Coffee Cuality Method (n = 56 experts).

The just-about-right ratings provide a first layer of justification for the quality ratings. Figure 5 shows their distribution for each JAR attribute and each coffee. They differed significantly among the coffees for all attributes (*p* < 0.05). The size of the JAR category (in green) compared with that of the “too low” (in red) and “too high” (in blue) combined categories clearly contrasts the coffees that received high quality ratings with those that did not in terms of color, roast level, flavor, acidity and body, thus providing important clues into the perceived quality of the coffees by the experts.

**Figure 6:**
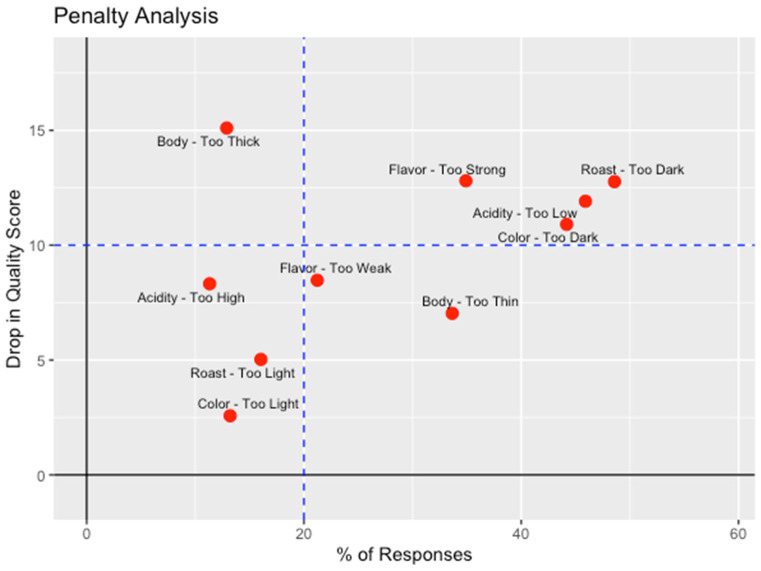
Penalty analysis from Coffee Cuality relating quality ratings to non-JAR ratings: drop in quality on the 100-point scale vs. percentage of experts who rated the JAR attributes of color, roast level, flavor, acidity and body as “too low” or “too high”, respectively. The upper right quadrant delineated by the dotted lines highlights those attributes for which ratings of “too low” or “too high” were selected by more than 20% of the experts and resulted in a drop in quality of more than 10 points on the 100-point scale (n = 56 experts).

But we must turn to the penalty analysis that relates quality to the JAR attributes being judged as “too low” or “too high” in Figure 6 to fully account for which JAR attributes mattered most in the determination of the quality of the coffees. Roast and color being judged too dark were the most detrimental to quality, with over 40% of the experts electing those categories and that resulting in a penalty of over 10 points on the 100-point quality scale. Other issues of significance were flavor being too strong and acidity being too low for 35% and 43% of the experts and resulting in 13- and 11-point penalties, respectively.

**Figure 7:**
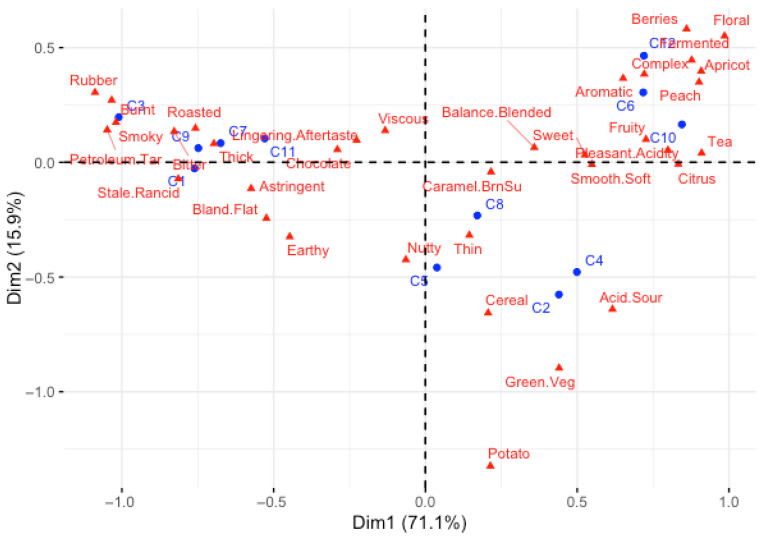
Sensory map of the coffees from Coffee Cuality: correspondence analysis of the matrix of CATA selections by the 56 experts across the 12 coffees showing both the coffees in blue and the sensory and holistic attributes in red in the first two dimensions. Cochran’s Q test was used to determine the significance of sensory and holistic attributes, *p* < 0.05.

The biplot from the correspondence analysis of the CATA selections then documents the specific sensory (and some holistic) attributes prevailing in the coffees (Figure 7). Those coffees rated high for quality, such as C6, C10 and C12, were found next to the floral, fruity, berries, peach, apricot, citrus, pleasant acidity, aromatic and complex attributes, whereas those that were rated low, such as C1, C3, C7, C9 and C11, were found next to the bitter, roasted, thick, astringent, rubber, burnt, petroleum/tar, smoky, stale and rancid attributes.

**Figure 8:**
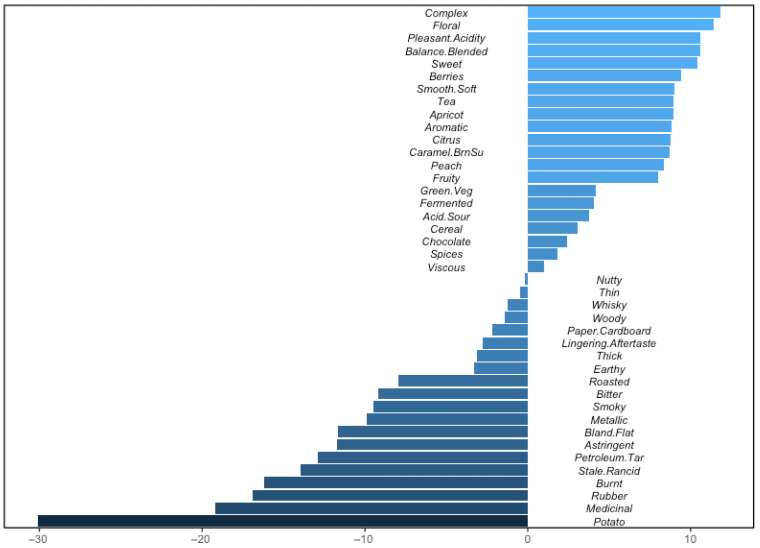
Penalty/lift analysis from Coffee Cuality relating quality ratings to CATA selections by the 56 experts for the 12 coffees. Those attributes to the right of the median are positive drivers of quality, whereas those to the left are negative drivers.

And the penalty/lift analysis relating the quality ratings to the CATA selections, in Figure 8, identifies the positive drivers of quality—complex, floral, pleasant acidity, balanced/blended, sweet, berries, smooth/soft, tea, apricot, aromatic, citrus, caramel/brown sugar, peach, green veggie, fermented, acid/sour, cereal, chocolate, spices and viscous, in this order—on one hand, and the negative drivers of quality—potato, medicinal, rubber, burnt, stale/rancid, petroleum/tar, astringent, bland/flat, metallic, smoky, bitter, roasted, earthy, thick, lingering aftertaste, paper/cardboard, woody, whisky, thin and nutty, in this order—on the other.

**Figure 9:**
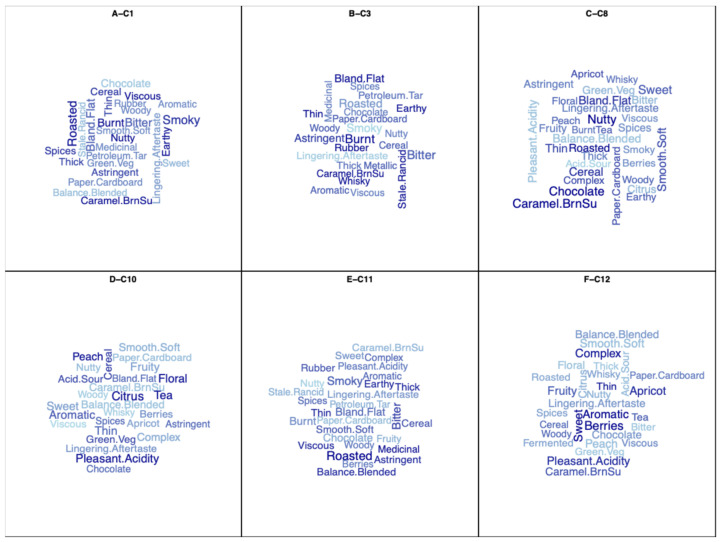
Word clouds derived from the CATA selections by the 56 experts for coffees C1 (**A**), C3 (**B**), C8 (**C**), C10 (**D**), C11 (**E**), and C12 (**F**). The size and boldening of the font are indicative of the frequency of selection of the attribute.

A visual, rather than statistical, and yet powerful and accessible confirmation of the sensory characteristics of the coffees can be found in the word clouds derived from the CATA selections. Those capture the sensory essence of each coffee for the viewer of that information. And again, they provide yet another means of documenting and justifying the quality ratings by the experts. Figure 9A–F shows the word clouds of coffees C1, C3, C8, C10, C11 and C12, chosen to represent and contrast some higher quality coffees with some lesser quality ones. The bigger the font for a given attribute, the more frequently it was selected by the experts to describe the coffee.

### 3.2. Focus Groups

Key learnings and supporting quotes from the focus groups are shown in Table 3.

Overall, participants found the method to be an improvement over the method they were used to, even though the idea of changing what was a well-entrenched coffee quality evaluation process (for which many had invested significant amounts of time and money in training and certification) was challenging for some. Upon having to justify their quality scores through the use of JAR ratings and CATA selections, most experts enjoyed those new elements of the scorecard and recognized their value (in documenting the overall quality rating). The main criticism was of the coffees we included in our research design, as some of the mainstream dark roasts in the set were considered too dark of a roast for a specialty coffee tasting. Indeed, a dark roast increases bitter and burnt flavors at the expense of the coffee’s natural flavor characteristics [32]. As a result, those dark roasts were systematically penalized and judged to not be “specialty” (by scoring well below 80 on the scale). That challenge aside, participants felt the logic of the sequence and flow of questions and scales in the Coffee Cuality scorecard made sense and was straightforward to navigate. Providing insights on the way the Coffee Cuality data is analyzed and sharing some of the outcomes of these analyses also was helpful for participants to understand the differences (and potential benefits) of the Coffee Cuality method over traditional ones.

The suggestions from the focus group participants were incorporated in the Coffee Cuality 2.0 scorecards that we now use for evaluating coffee quality with experts (Figure 2 for cupping and drip coffee and Supplementary Figures S1 and S2 for espresso and cold brew, respectively).

## 4. Discussion

The Coffee Cuality Method allowed for a comprehensive explanation and validation of the quality ratings by the experts based on (1) the mean quality ratings of the coffees, which can be treated as definitive since there was no substantial segmentation of the experts based on their quality ratings, (2) the correspondence analysis biplot of the CATA selections, (3) the word clouds derived from those selections for each coffee to identify which attributes characterized the coffees rated high in quality and those rated low, (4) the penalty/lift analysis of the CATA selec-tions to identify the positive and negative drivers of quality, (5) JAR ratings and (6) their penalty analysis to see how the JAR attributes were on or off target as far as the quality of the coffees was concerned.

Let us examine two of the coffees in the design to illustrate the above claims. Coffee C10 received the highest mean quality rating (Table 2) and has most of the expert quality vectors in Figure 3A,B pointing at it. The majority of the experts rated the five JAR attributes of color, roast level, flavor, acidity and body as just right for C10 (Figure 5). Its position in the sensory map on Figure 7 associates C10 with fruity and floral attributes, balance and complexity. This is confirmed by C10’s word cloud in Figure 9D in which peach, citrus, tea, floral and pleasant acidity feature prominently—all features that were highlighted in the descriptive analysis of this Ethiopia light roast coffee by Batali et al., 2022 [33]. By contrast, Coffee C3 received the lowest quality rating in the design and appears away from the quality vectors of the experts in Figure 3A,B. Almost all the experts judged it to be too roasted, too dark in color, too intense in flavor, not acidic enough, and either too thin or too thick in body, clearly signaling that the coffee was off balance because of excessive roasting (Figure 5). On the sensory map derived from the CATA selections (Figure 7), C3 is characterized by burnt, rubber, smoky, petroleum/tar and bitter attributes. And its word cloud singles out rubber, stale, rancid, burnt, astringent and bland/flat as its key features. In short, Coffee Cuality thoroughly documents the reasons why a coffee received a high or a low quality rating on the 100-point scale.

That the experts would use a broader part of the 100-point quality scale when evaluating the coffees in the context of the Coffee Cuality method and would in turn be more discriminating among the coffees (Table 2) is interesting since after all, both their conventional or preferred method and Coffee Cuality use a 100-point scale. It signals both a restraint when using a conventional method and a desire to expand quality ratings (particularly upward), both expressed in the focus groups that we conducted (Table 3).

In this research, we also verified the hypothesis that experts could be aligned in their concept of sensory quality. And indeed, the agreement among our experts as to which coffees were of higher quality and which were of lesser quality was very high. This is shown by the proximity of (or the small angle among) their quality vectors in the quality maps (Figure 3A,B) or the clustering of most of the experts together in the quality clustering dendrograms (Figure 4A,B), with only a few experts outside the main cluster. Even those remain close to the majority on the quality maps, though. So, we can confirm that with the extensive training they receive, coffee experts can consistently rate the quality of coffee following set industry criteria. Having made this statement, this does not solve the problem we set to solve, which is the lack of documentation and justification of those quality ratings with conventional methods

While the Coffee Cuality™ method is at its most efficient when multiple experts evaluate each coffee, as the afforded statistical power allows for the full suite of statistical analyses to be performed (i.e., quality mapping, penalty analysis relating quality ratings with JAR ratings, penalty/lift analysis relating quality ratings with CATA selections, and word analyses), it can also be used with one expert and the one completed scorecard with the lone expert’s quality rating, JAR ratings, CATA selections and open comments then becomes the definitive evaluation of the coffee. Such a lone expert evaluation is common in the trade. But in this instance again, Coffee Cuality adds valuable information to the quality rating provided by the expert.

This research confirmed the value as well of combining quantitative and qualitative approaches—for understanding the sensory appeal of coffee to experts in this case. The key learnings from the focus groups provided yet another layer of information on top of the quantitative measures in the Coffee Cuality scorecard and their analyses.

There are some differences worth highlighting between our Coffee Cuality method and the SCA’s new Coffee Value Assessment (CVA) method. Whereas Coffee Cuality™ uses JAR ratings, CATA selections, open comments and their statistical analysis to justify an overall quality rating on a 100-point scale, the CVA consists of a descriptive assessment first, including roast level, that combines intensity ratings of fragrance, aroma, flavor, aftertaste, acidity, sweetness, and mouthfeel, with CATA selections, including defects, and second an “affective” assessment which actually is an “impression of quality” rating from 1 indicating “extremely low” to 9 indicating “extremely high” quality [28].

Future research should compare the respective outcomes and merits of the SCA’s new Coffee Value Assessment Method [28], The Espresso Protocol (TEP) [29], and Coffee Cuality, which came first (in 2020). They share elements and yet have evolved in different enough directions to warrant such a comparison.

Despite the undoubtable value of (objective) instrumental measurements for the quantification of the sensory attributes of coffee and the prospect of using those for the assessment of coffee quality [13], they still represent a substantial investment in equipment and personnel that may not be able to replace the figure and role of the “expert” who must conduct the sensory evaluation of the coffee for trading, marketing and other purposes. And yet, with machine learning prediction of the sensory profile of coffee from instrumental measures well under way, we may be getting closer to the successful prediction of coffee quality from such instrumental measurements of coffee sensory properties.

## 5. Conclusions

This study demonstrates that Coffee Cuality™ is effective in providing first an overall quality rating of the coffee and then deconstructing and justifying that quality rating through the use of JAR ratings, CATA selections, open comments and their statistical analyses. Even though it represented a change at the time of its introduction, the experts in this study were able to use (and even value, for most) this new approach, as documented in the key learnings from our focus groups. With proper training on the basics and mechanics of the method, expert coffee tasters could use Coffee Cuality™ to assess the sensory quality of coffee, for trading or marketing purposes, and that could be with one expert and one scorecard at a time or multiple expert evaluations, in which case the full battery of statistical analyses could be applied to deconstruct and justify the quality rating of the coffee.

## Figures and Tables

**Table 1:** Commercial and specialty coffees used, with roast level and whole and ground bean Agtron Gourmet scores.

Brand/Origin	Type	Roast Level	Agtron Gourmet Score—Whole Bean	Agtron Gourmet Score—Ground
Starbucks French Roast	Commercial	Dark	25.4	25.2
Peet’s Major Dickason Blend	Commercial	Dark	45.3	43.9
Kirkland Columbian Supremo (Blind)	Commercial	Dark	40.4	44.3
Ethiopia Natural Denbi Uddo	Specialty	Medium	62.7	70.5
El Salvador Cerro Las Ranas Honey	Specialty	Light	58.74 ± 1.40	68.18 ± 0.88
El Salvador Cerro Las Ranas Honey	Specialty	Medium	49.64 ± 1.15	53.40 ± 1.15
El Salvador Cerro Las Ranas Honey	Specialty	Dark	36.78 ± 1.49	35.92 ± 1.58
Ethiopia Guji Washed Organic	Specialty	Light	56.96 ± 0.79	68.20 ± 0.72
Ethiopia Guji Washed Organic	Specialty	Medium	49.46 ± 1.33	54.16 ± 1.11
Ethiopia Guji Washed Organic	Specialty	Dark	36.82 ± 1.93	34.98 ± 1.94
Sumatra Fair-Trade Organic Takengon	Specialty	Light	56.14 ± 0.74	66.96 ± 1.34
Sumatra Fair-Trade Organic Takengon	Specialty	Medium	49.24 ± 0.93	53.48 ± 1.28
Sumatra Fair-Trade Organic Takengon	Specialty	Dark	38.12 ± 0.69	35.62 ± 0.46

**Table 2:** Mean coffee quality ratings on a 100-point scale and standard deviations with the conventional and Coffee Cuality methods. Means sharing letter superscripts are not significantly different, but those not sharing a letter superscript are, per Fisher’s LSD (*p* > 0.05).

Coffee	Conventional and/or Preferred Method	Coffee Cuality
C1	70.87 ± 18.20 ^bc^	67.68 ± 19.36 ^e^
C2	75.10 ± 18.36 ^ab^	75.87 ± 13.66 ^cd^
C3	66.30 ± 17.82 ^c^	59.04 ± 23.24 ^f^
C4	74.75 ± 19.50 ^ab^	78.10 ± 10.82 ^bc^
C5	75.95 ± 18.40 ^ab^	78.37 ± 7.40 ^bc^
C6	79.60 ± 18.36 ^a^	84.62 ± 5.17 ^a^
C7	72.60 ± 16.85 ^bc^	69.59 ± 18.64 ^e^
C8	75.68 ± 18.26 ^ab^	78.61 ± 8.25 ^bc^
C9	71.35 ± 17.31 ^bc^	67.30 ± 20.34 ^e^
C10	79.52 ± 18.18 ^a^	82.71 ± 10.31 ^ab^
C11	72.73 ± 17.78 ^abc^	71.14 ± 18.10 ^de^
C12	79.61 ± 18.25 ^a^	79.83 ± 18.32 ^abc^

**Table 3:** Focus group key learnings and supporting quotes from the experts (n = 18) regarding their experience with the Coffee Cuality Method.

About specialty coffee assessment	
	**Some historical background and views on current grading methods and practices provided important context and confirmed the need for an evolution.**
1	“When the original SCA form came out, the industry first revolted against it. And we have all resisted at various stages along the way, but it naturally works very well in terms of creating a discipline and a standard format for people to understand scores and other components of cupping across geographical and social barriers.”
2	“The original intent of the scores was to help the farmers get their coffee on the map.”
3	“With the SCA scorecard, we just give our opinion about the coffees, we’re not measuring anything except our own personal [opinion]; do we like it or do we not like it?”
4	“[The 100-point scale] turned into something like school grading… this was in the A range, this in the B range, etc.”
5	“Everything that’s dark is going to be called out-of-spec [on the JAR scale] because that’s the paradigm I am working with.”
6	“You must have 3 Q graders grade a sample of green coffee and then it would be certified as specialty or not.”
7	“Yes, I take major issue with the fact that we use 10 points out of 100, but it serves its purpose.”
8	“Break away from just sharing a number with the consumer; to them it doesn’t really matter, and to us, it doesn’t make any sense.”
9	“It is very difficult to score from attributes [in reference to SCA scorecard]. People cheat constantly.”
10	“In coffee, we are a bit of an echo chamber and we are stating scores that our colleagues would approve of.”
11	“An industry tends to be fairly unified in their opinion of things.”
12	“We tend to convey to each other quite well, but not to the greater public. Ther eis a need for an approach that helps communication with the consumer.”
13	“A lot of people in the industry tend to underestimate how good a consumer can be at perceiving quality.”
b. About Coffee Cuality	
	**Documenting the overall quality score—a key benefit of Coffee Cuality.**
1	“Filling all the categories made me feel more objective.”
2	“The argument your [Coffee Cuality] system could have is, OK, it’s specialty, but what other characteristics can I explain in the supply chain, as a buying value proposition, is there fruitiness to it? Is there some other way to say yes, it’s specialty, but here is why.”
3	“I can see it [CATA] adding some additional clarity or value or delineation, so now it’s not just an 80 or 84, its an 80 or 84 with these characteristics.”
4	“I felt I had an opportunity to explore and perhaps reward coffees in a way that the SCA score does not allow for.”
5	“This new form tries to communicate what a coffee is, what it does well, and what it doesn’t.”
6	“All the different things after the overall score I used to complement it, like the flavors, the body, the defects.”
	**The Coffee Cuality scorecard was user-friendly to most.**
1	“I thought the form was very intuitive because we use similar check boxes, similar questions.”
2	“The simplicity of the form is attractive.”
3	“This was fascinating.”
	**Change is hard.**
1	“When cupping coffees, I evaluate an entire table (5), and I do four passes, so doing one sample at a time was a challenge.”
2	“This was very challenging for someone comfortable with and used to scoring with the SCA’s [scoring system].”
3	“It is very difficult when you move to another medium; some of your skills translate but the tools you use are very different.”
	**Many experts viewed Coffee Cuality as an instrument that speaks to the consumer.**
1	“It seems like the form was set up to be consumer facing.”
2	“I think it is great to create a common language for the consumer [and the producer]; and one the buyer can link to so they can sell more coffee.”
3	“I think it’s great if we can continue to educate consumers and make them comfortable with coffee language.”
4	“That’s where the Q grading system to date is still on an uphill battle in communicating those coffee scores to the consumer.”
5	“I think it’s great if we can continue to educate consumers and make them comfortable with coffee language.”
	**The choice of JAR and CATA attributes drew mostly positive comments, but also some constructive criticism.**
1	“I really liked the JAR scales.”
2	“I actually loved the check-all-that-apply.”
3	“I enjoyed that I could quickly identify a couple of major flavor profiles.”
4	“I like a lot of what this project is doing because of the potential for statistical analysis, in a way that we can kind of certify those descriptors.”
5	“The set [of CATA attributes] was missing a lot of good attributes in the coffee; balancing the stack would be good.”
6	“It may be good to separate negative characteristics [in the CATA section] versus defects; the word ‘defect’ means something very specific to Q graders.”
**7**	“Distinguish between unpleasant notes and defects.”
**8**	“As a Q-grader, you look at many other things that can creep into a score.”
**9**	“Scoring those attributes on 5 to 6 coffees is a very pleasant experience; when you get to 10, it’s actually a hindrance to the sensory experience of the coffee.”
**10**	“The form is pretty good except I found myself searching for flavors I use a lot.”
**11**	“Coffee Cuality is much more right brain in terms of its abstract approach to coffee.”
	**Coffee Cuality was confusing to some and there is a need for training on how to use the [Coffee Cuality] scorecard.**
**1**	“It is very difficult to take a new methodology which has an objective component like a numerical score and a subjective component to it.”
**2**	“There is a major disconnect in the form.”
**3**	“I just transferred the scores [from the SCA scale to the Coffee Cuality scale].”
**4**	“It is still a matter of individual choice. So, calibrating with people is very challenging.”
**5**	“It allowed me to expand the range of scores, and a 50 would not necessarily be bad.”
	**Coffee Cuality, used the right way, could be a better way to convey quality (and market coffee) to the consumer.**
**1**	“We don’t match the Wine Spectator, which can go up to 96; we are very conservative.”
**2**	“We tend to score between 85 and 88 unless something is really good.”
**3**	“I don’t know how we move the industry that way, but for all of us who have been grading [coffee], 95 is A+; let’s get the coffee scores in this area, I think we’ll sell more.”
	**Coffee Cuality is useable across the entire coffee chain.**
**1**	“There is value in a method and language that the entire coffee chain could relate to.”
**2**	“I like the idea of going to different parts of the industry with this.”
	**Miscellaneous**
	“The form is too long and cumbersome. The same content could be scaled down and fit on one page.”
c. About the study design and protocol	
	**The sample set was somewhat inappropriate as it included dark roasts not typically evaluated by specialty coffee cuppers, and 12 coffees were too many to evaluate in one sitting for several experts.**
1	“Coffee samples were not the standard we use”
2	“Some of these were really, really dark, so I couldn’t score any kind of taint or fault.”
3	“I overrode your instructions and divided the samples into two sets because that is pushing the limits.”
4	“10 coffees max; beyond, it’s like I just got to get through this.”
5	“We work with different roasts, and we would do all the light, all the medium, and all the darks because if you have a mishmash, the darker roasts will always taste burnt and ashy.”
6	“These coffees were challenging in terms of their roast profile.”
7	“Contrast effects are at play when evaluating so many coffees.”

## Data Availability

The original contributions presented in this study are included in the article and Supplementary Material. Further inquiries can be directed to the corresponding author.

## Supplementary Materials

The following supporting information can be downloaded at https://www.mdpi.com/article/10.3390/foods15040678/s1: Figure S1: Coffee Cuality™ 2.0 scorecard for espresso; Figure S2: Coffee Cuality™ 2.0 scorecard for cold brew.
