# Supplementary material for: Validation of the Coffee Cuality™ Method for the Expert Assessment of Coffee Sensory Quality
Source: Foods. 2026 Feb 12;15(4):678. doi: 10.3390/foods15040678 (PMC12938992; doi:10.3390/foods15040678)
Supplement: Supplementary file 1 [file foods-15-00678-s001.zip › foods-4123719-supplementary.pdf]

Coffee Cuality™ 2.0 Scorecard – Espresso

Coffee Code _______________ Judge Name _______________ Date ____________________

Overall quality

Please rate the **overall quality** of the coffee on a 100-point scale________________

Adequacy of select attributes – Just-About-Right (JAR) scaling

Appearance - Please indicate how you feel about the appearance of the coffee (circle your answer):

- The **volume of the crema** is:

Much too low | Somewhat too low | Just about right | Somewhat too high | Much too high

- The **consistency of the crema** is:

Much too thin | Somewhat too thin | Just about right | Somewhat too thick | Much too thick

- The **color of the coffee** is:

Much too light | Somewhat too light | Just about right | Somewhat too dark | Much too dark

Temperature - Please indicate how you feel about the temperature of the coffee (circle your answer):

Much too cold | Somewhat too cold | Just about right | Somewhat too hot | Much too hot

Roast Level - Please indicate how you feel about the roasting of the coffee (circle your answer):

Much too light | Somewhat too light | Just about right | Somewhat too dark | Much too dark

Flavor (Aroma, Taste & Trigeminal) – Please indicate how you feel about the **flavor** of the coffee (circle your answer):

Much too weak | Somewhat too weak | Just about right | Somewhat too strong | Much too strong

Acidity - Please indicate how you feel about the acidity of the coffee (circle your answer):

Much too low | Somewhat too low | Just about right | Somewhat too high | Much too high

Bitterness - Please indicate how you feel about the bitterness of the coffee (circle your answer):

Much too low | Somewhat too low | Just about right | Somewhat too high | Much too high

Body - Please indicate how you feel about the body of the coffee (circle your answer):

Much too thin | Somewhat too thin | Just about right | Somewhat too thick | Much too thick

Coffee Code _______________ Judge Name _______________ Date ____________________

Description of the coffee – Check-All-That-Apply (CATA)

From the list of attributes/features in each category below, please circle all that apply to this coffee:

Flavor (Aroma, Taste & Trigeminal)

Roasted | Green/Vegetative | Cereal/Grain | Tea | Nutty | Chocolate | Spices

Fruity* | Earthy | Whisky | Floral | Smoky | Caramel/Brown Sugar

Citrus

Peach/Apricot | Acid/Sour | Bitter | Sweet | Lingering Aftertaste

Berries

**You may circle the general fruity descriptor and/or any of the specific fruity descriptors*

Body/Mouthfeel

Thick | Thin | Viscous | Astringent | Smooth/Soft

Holistic/Hedonic

Aromatic | I like it! | Bland/Flat | Complex | Balanced/Blended

Defects

Stale/Rancid | Paper/Cardboard | Fermented | Burnt | Rubber

Medicinal | Petroleum/Tar | Woody | Metallic | Potato

Comments:

____

____

© 2023 2022 2021 2020 2019 Jean-Xavier Guinard

Figure S1- *Coffee Cuality™ 2.0 scorecard for espresso*

Coffee Cuality™ 2.0 Scorecard – Cold Brew

Coffee Code _______________ Judge Name _______________ Date ____________________

Overall quality

Please rate the **overall quality** of the coffee on a 100-point scale________________

Adequacy of select attributes – Just-About-Right (JAR) scaling

Appearance - Please indicate how you feel about the appearance of the coffee (circle your answer):

- The **color** is:

Much too light | Somewhat too light | Just about right | Somewhat too dark | Much too dark

Temperature - Please indicate how you feel about the temperature of the coffee (circle your answer):

Much too cold | Somewhat too cold | Just about right | Somewhat too warm | Much too warm

Roast Level - Please indicate how you feel about the roasting of the coffee (circle your answer):

Much too light | Somewhat too light | Just about right | Somewhat too dark | Much too dark

Flavor (Aroma, Taste & Trigeminal) – Please indicate how you feel about the **flavor** of the coffee (circle your answer):

Much too weak | Somewhat too weak | Just about right | Somewhat too strong | Much too strong

Acidity - Please indicate how you feel about the acidity of the coffee (circle your answer):

Much too low | Somewhat too low | Just about right | Somewhat too high | Much too high

Bitterness - Please indicate how you feel about the bitterness of the coffee (circle your answer):

Much too low | Somewhat too low | Just about right | Somewhat too high | Much too high

Body - Please indicate how you feel about the body of the coffee (circle your answer):

Much too thin | Somewhat too thin | Just about right | Somewhat too thick | Much too thick

Coffee Code _______________ Judge Name _______________ Date ____________________

Description of the coffee – Check-All-That-Apply (CATA)

From the list of attributes/features in each category below, please circle all that apply to this coffee:

Flavor (Aroma, Taste & Trigeminal)

Roasted | Green/Vegetative | Cereal/Grain | Black Tea | Nutty | Chocolate | Brown Spice

Fruity* | Grassy | Floral | Dried Fruit | Caramel/Brown Sugar | Molasses | Whisky

Citrus

Peach/Apricot | | | | | | Lingering Aftertaste

Berry | Earthy | Broth | Acid/Sour | Bitter | Sweet

**You may circle the general fruity descriptor and/or any of the specific fruity descriptors*

Body/Mouthfeel

Thick | Thin | Viscous | Astringent | Smooth/Soft

Holistic/Hedonic

Aromatic | I like it! | Bland | Complex | Balanced

Defects

Stale/Rancid | Paper/Cardboard | Fermented | Burnt/Smoky/Ash | Rubber

Medicinal | Petroleum/Tar | Woody | Metallic | Potato

Comments:

____

____

© 2023 2022 2021 2020 2019 Jean-Xavier Guinard

Figure S2- *Coffee Cuality™ 2.0 scorecard for cold brew*
